# Nurse-led hypertension management was well accepted and non-inferior to physician consultation in a Chinese population: a randomized controlled trial

**DOI:** 10.1038/s41598-018-28721-2

**Published:** 2018-07-09

**Authors:** Benjamin Hon Kei Yip, Eric Kam Pui Lee, Regina Wing Shan Sit, Carmen Wong, Xue Li, Eliza Lai Yi Wong, Martin Chi sang Wong, Roger Yat Nork Chung, Vincent Chi ho Chung, Kenny Kung, Samuel Yeung shan Wong

**Affiliations:** JC School of Public Health and Primary Care, The Chinese University of Hong Kong, Hong Kong SAR, China

## Abstract

The objective of this study is to evaluate if nurse-led repeated prescription (NRP) could ensure non-inferior disease control and would be accepted in Chinese patients with controlled hypertension (HT) in primary care clinics. A 12-month follow-up non-inferiority randomized trial was conducted. The non-inferior margins for systolic and diastolic blood pressure were 6.6 mmHg and 3.7 mmHg, respectively. Eligible patients (>18 years of age) with HT were randomized to the NRP and usual care (UC) groups for their regular clinical follow-up. We used ANCOVA to study the difference-of-difference of the blood pressures between the two groups. The levels of patient acceptance and experience of NRP were assessed by the observed opt-out rate and a qualitative analysis. We found no statistically significant differences in BP blood pressure between the NRP (N = 194) and UC (N = 199) groups. Only 4 of the participants in the NRP group opted out due to a preference for assessment by a physician. The interviewed participants (N = 12) felt positive about NRP, because they experienced more relaxed communication with the nurse and believed that the eligibility to join the NRP program was an indication of optimal BP control. We observed no adverse events. The findings show that NRP was well accepted and found to be non-inferior to physician consultation for HT management.

## Introduction

Hypertension (HT) is the most common chronic disease in developed countries^1^ – around 26 to 46 percent of adults suffer from hypertension^2,3^. HT is responsible for 7.6 million deaths worldwide every year and around half of the coronary artery diseases and stroke are attributed by HT^4^. The increasing prevalence and huge patient volume of HT put heavy burdens on healthcare systems and threatens global economic growth^3^.

Repeat prescriptions (RP) for patients with well-controlled chronic disease(s), including HT, is an order for repeat medication to be supplied to a patient without direct physician consultation^5^. RPs might be convenient for patients, allowing them to share responsibility for their medical care, significantly reducing clinic attendance^6^. From operational perspective RPs might also improve access to medicines, better utilization of economic and human resources, as well as reducing patient waiting times and fragmentation of care^7^.

The concept of RP was first introduced in the 1970s and is still practicing in UK, Australia, and some US states. In the UK, once an agreement between a prescribing physician, patient, and supplementary prescriber (most commonly nurses or pharmacists with relevant postgraduate training) is reached, the supplementary prescriber can prescribe medications to the patient based on a specific management plan formulated by the prescribing physician^7–9^. In the UK, 77% of the drugs prescribed in primary care were RPs in 2011, and 43% of the population was prescribed with at least 1 medication by RP^10^. Relatively, NRP remains as a new practice in Asia. In Japan and Korea, the nurse-led chronic disease management is mainly still focusing on education and psychology care with no prescription right^11,12^. Recently, Singapore government expanded the NRP approach in non-communicable diseases management based on some positive results from a few pilot trials^13^. Whether NRP is acceptable and feasible in China is still unknown.

In Hong Kong, most patients with chronic diseases (>87%) are followed up in the public sector, as a service provided by the Hospital Authority through general outpatient clinics (GOPCs)^14^. Patients with chronic diseases make around 60% of GOPC attendances^15^, with HT being the most common^14^. For every 3–4 months, HT patients had to revisit the clinic to receive their prescribed drugs, irrespective of their blood control status. This 3-month follow-up period limits a number of medications dispensed per prescription, controlling the cost per prescriptions. However, this is at the expense of the physician’s consultation which could be used for patients with other more pressing complaints^16^. What’s more, in China, patients seem to have a preference to go to physicians for medical care which they believe to be more reliable^17^. In this condition, a significant number of patients have made the hospitals overstraining^18^.

The World Health Organization (WHO) encourages building community-based healthcare teams to enhance access to healthcare; this may benefit China, where the physician-to-population ratio is far below the WHO standard^19^. Furthermore, HT is highly prevalent in China^20^ and vascular diseases, most of which are contributed by HT, are the most common cause of death in China^21^. NRPs may partly solve the predicted shortage of physicians caused by this large burden of HT^22^.

RPs led by a nurse is one way to achieve this goal and might reduce physician burdens. It is, however, not known if nurse-led RPs (NRP) is feasible and acceptable in the Chinese population. Our study aimed to determine if NRP in a GOPC could result in non-inferior disease control and patient acceptance when compared to conventional HT management by physicians in patients with well-controlled HT. We hypothesized that patients in the NRP group would achieve comparable disease control regarding systolic (SBP) and diastolic blood pressure (DBP) over a one-year period and that NRP would be acceptable to these patients.

## Results

**Figure 1:**
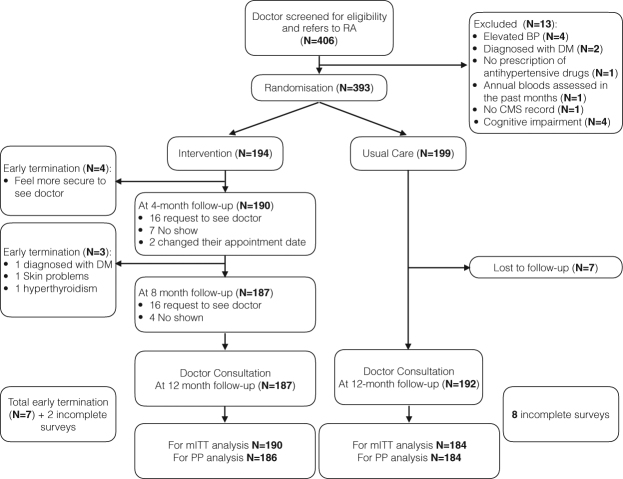
The CONSORT flow diagram, mITT(modified ITT analysis), PP(per-protocol analysis).

Of the 406 participants, 194 and 199 were randomized to the NRP and UC groups, respectively, after exclusions (Fig. 1). The opt-out in the NRP group was minor; 4 patients felt more secure under the care of their case physicians or were referred to their case doctors for other diseases. In the UC group, 7 participants did not attend follow-up visits. Consequently, the mITT analysis was based on 194 and 192 patients from the NRP and UC groups, respectively. The PP analysis was based on 187 and 192 participants from the NRP and UC groups, respectively.

### Baseline characteristics

The average age of the participants was 63.5 years. Most were women, married, had a primary to secondary school education, and were non-smokers. About half had an HT diagnosis of >7 years (Table 1). Drug compliance was good, with an average MMAS-8 score of 7.4 in both groups. Consistent with usual prescribing practice in GOPCs, calcium channel blockers were the most common anti-hypertensive medication class used, followed by beta-blockers^23^. We detected no significant differences in demographic variables, clinical characteristics, and primary/secondary outcomes between the two groups (Table 1).

**Table 1:** Demographic characteristics of participants at baseline (n = 393) by intervention.

Items	Usual Care (N = 199)	NRP (N = 194)	*P* value*
SBP (mmHg), mean (sd)	123.4 (10.8)	123.8 (9.7)	0.666
DBP (mmHg), mean (sd)	72.3 (9.0)	73.1 (9.4)	0.429
Age, mean (sd)	62.9 (8.3)	64.0 (9.1)	0.197
Gender = Male, n (%)	72 (36.2)	85 (43.8)	0.149
Marital Status, n (%)			0.347
Single	15 (7.5)	7 (3.6)	
Married	149 (74.9)	151 (77.8)	
Widowed	24 (12.1)	22 (11.3)	
Separated	11 (5.5)	14 (7.2)	
Education level, n (%)			0.330
Illiterate	21 (10.8)	21 (10.8)	
Primary 1 to 6	79 (40.5)	90 (46.4)	
Secondary 1 to 7	85 (43.6)	79 (40.7)	
Tertiary or above	10 (5.1)	4 (2.1)	
Smoking status, n (%)			0.301
Current	13 (6.5)	14 (7.2)	
No	167 (83.9)	152 (78.4)	
Past	19 (9.5)	28 (14.4)	
Year since HT diagnosis, n (%)			0.338
<2 years	35 (17.7)	32 (16.6)	
2–7 years	63 (31.8)	75 (38.9)	
>7 years	100 (50.5)	86 (44.6)	
Patient Enablement Index, mean (sd)	3.1 (2.9)	2.71 (2.9)	0.175
MMAS-8	7.4 (1.1)	7.4 (1.1)	0.411
Anti-hypertensive prescriptions			0.081
ACEIs or ARBs	26 (13.1)	42 (21.6)	
Beta-blocker	68 (34.2)	56 (28.9)	
Calcium blocker	145 (72.9)	149 (76.8)	
Diuretics	21 (1.0)	20 (10.3)	
Others	2 (1.0)	8 (4.1)	

^*^p value was based on independent sample t-test for continuous variables and chi-square test for categorical variables.

NRP: Nurse led repeated prescription, sd: standard deviation, SBP: Systolic Blood Pressure, DBP: Diastolic Blood Pressure, HT: hypertension, MMAS-8: 8-Item Morisky Medication Adherence Scale, ACE: Angiotensin converting enzyme inhibitors (Lisinospril), ACR: Angiotensin Receptor Blockers (Losartan), Beta blocker includes atenolol, metoprolol, Calcium blockers includes amlodipine, nifedipine, Diuretics includes hydrochlorothiazide, moduretic, Others prescriptions includes methylopa and prazosin.

### Primary outcome (blood pressure)

**Figure 2:**
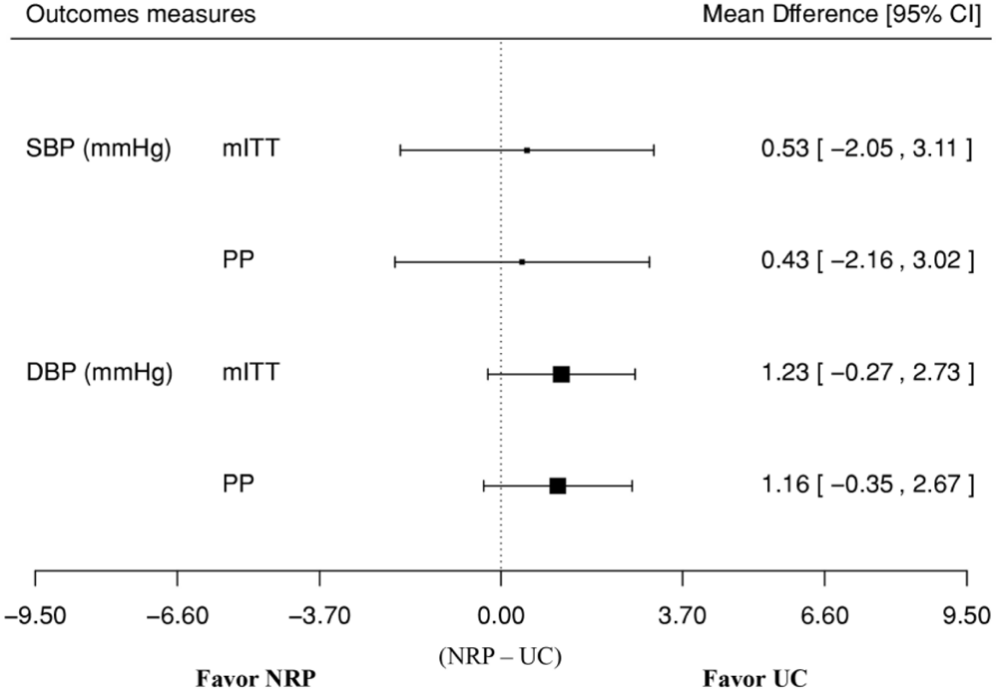
Estimated mean difference in systolic blood pressure(SBP) and diastolic blood pressure (DBP) between intervention group (nurse led repeated prescription, (NRP) and control group (usual care, UC), by modified intention-to-treat (mITT) and pre-protocol analysis.

The baseline-adjusted mITT and PP mean differences (NRP - UC) for SBP were 0.53 mmHg (95% CI −2.05 to 3.11 mmHg) and 0.43 mmHg (95% CI −2.16 to 3.02), respectively (Fig. 2). For DBP, the baseline-adjusted mITT and PP mean differences were 1.23 mmHg (95% CI −0.27 to 2.73 mmHg) and 1.16 mmHg (95% CI −0.35 to 2.67), respectively. Since none of these estimates had a lower bound of the 95% CI that crossed the non-inferiority margin for SBP (6.6 mmHg) and DBP (3.7 mmHg), NRP was found to be non-inferior to UC.

### Patient enablement and drug compliance

PEI scores at 12 months were very similar between the two groups (eFigure 1), irrespective of the type of analysis. Patients in the NRP group had a slightly lower MMAS-8 score than those in the UC group (mean difference −0.22; 95% CI −0.44 to 0.01); however, this was not statistically significant (p = 0.0582 and p = 0.0746 for mITT and PP, respectively).

### Healthcare utilization and anti-hypertensive prescriptions

We observed no changes in the type and dosage of antihypertensive medications prescribed in more than 90% of the patients (Table 2). Healthcare utilization, including extra GOPC visits, private clinic visits, hospitalization and AED admissions, and changes in anti-hypertensive prescriptions were not statistically different between the two groups.

**Table 2:** Health care utilization and anti-hypertensive prescription at the end of follow-up (12 month).

Items	Usual Care (N = 184)	NRP (N = 190)	*P* value*
Private hospitalization (%)			0.493
0	177 (96.2)	185 (96.4)	
1	6 (3.3)	3 (1.6)	
>1	1 (0.5)	2 (1.1)	
Private clinic visit (%)			0.192
0	86 (46.7)	82 (43.2)	
1–10	39 (21.2)	31 (16.3)	
>10	59 (32.1)	77 (40.5)	
GOPC visit (%)			0.234
0–5	121 (65.8)	139 (73.2)	
6–10	57 (31.0)	44 (23.2)	
>10	6 (3.3)	7 (3.7)	
A&E visit (%)			0.928
0	152 (82.6)	158 (83.2)	
1	27 (14.7)	28 (14.7)	
>1	5 (2.7)	4 (2.1)	
Public hospitalization %)			0.582
0	157 (85.3)	168 (88.4)	
1	18 (9.8)	12 (6.3)	
2	5 (2.7)	7 (3.7)	
>2	4 (2.2)	3 (1.6)	
SOPD visit (%)			0.797
0	117 (63.6)	123 (64.7)	
1	24 (13.0)	29 (15.3)	
2	19 (10.3)	15 (7.9)	
>2	24 (13.0)	23 (12.1)	
Type of anti-hypertensive (%)			0.226
Reduce type of anti-hypertensive	4 (2.2)	2 (1.1)	
No Change	174 (94.6)	186 (97.9)	
Add a new anti-hypertensive	6 (3.3)	2 (1.1)	
Change dosage (%)			0.055
No changed	162 (88)	179 (94.2)	
Changed	22 (12)	11 (5.8)	

*p value was based on chi-square test.

RP: Nurse led repeated prescription, GOPC: General out-patient clinic, SOPD: Special out-patient clinic, A&E: Accident & emergency department.

### Qualitative analysis

We interviewed 12 participants of the NRP group. All participants expressed positive comments about NRP, with the following three main themes (eTable 2 for detailed results):

#### Theme 1: Reasons for high acceptance of NRP

Patients were willing to join the program due to their perceived control of HT; the competency of the nurse; and the additional support from the nurse.

#### Theme 2: Interaction with the nurse

All interviewees evaluated the NRP clinic positively; none reported having problems with the program. Compared to usual practice with physician’s consultation, they felt that they had better communication with the nurse; were more relaxed when visiting the nurse, and that time was managed better.

#### Theme 3: Value of the NRP clinic

All interviewees were willing to continue in the NRP clinic as long as physician consultations would be arranged periodically and/or if there was good communication between the doctor and the nurse.

The results of qualitative study fit very well with our quantitative restuls (Table 3). Although there were no complaints, some participants expressed opinions on the possible downsides of NRPs. One participant reported that he/she wished to be seen by a physician at the time of NRP in the case of other physical problems. Another participant stated that NRP did not allow for changes in HT medication doses (nurses have prescription right in Hong Kong).

**Table 3:** Agreement between quantitative and qualitative findings.

Quantitative findings	Qualitative findings
Very low dropout rate in nursing clinic group	Better communication with nurse
	Feeling more relaxed in nurse clinic
	Save time for doctors
	Willing to find alternate help for new acute problems
	Willing to pay more for nursing clinic
Participants blood pressure was well controlled. Most did not have drug titration in the study period	Perceived very good control of blood pressure
Outcome measures were very similar between NRP group and UC group	Lack of perceived difference between nurse and doctors

## Discussion

Our findings suggest that NRP is non-inferior to UC for BP management in patients with controlled HT. We found similar blood pressure outcomes in both groups, similar medication changes and no adverse events in both groups. The two groups also have a comparable proportion of attendance to alternative sources of medical attention. NRP seemed to be accepted by patients, as only 4 of 197 patients opted out due to a preference to be assessed by a physician.

Our qualitative analysis revealed similar findings as previous studies^5,7,9,24^. Patients believed that NRP might reduce physician workload and that doctors and nurses should communicate adequately and share responsibility. We found that some patients were even willing to pay more to see a nurse than a physician (see supplementary). In contrast to previous studies, patients in our study were more ready to arrange another medical consultation if new acute problems arose^5^.

One interesting recurring theme revealed in our qualitative analysis was that patients were willing to seek alternative help for new acute problems, as the nurse could not provide other drugs; yet, there was no significant difference in healthcare utilization between the two groups. It is possible that in our sample the need for other prescriptions was small because of the well-controlling of their hypertension, and consequently, physician consultation was not necessary. Thus, our findings may not be replicable in a sample with more complicated health conditions. Potentially, the medical workforce may be allocated more efficiently in this way, since physician consultation can be set aside for acute and severe patients.

Our data suggest that NRP clinics by allied healthcare professionals should be further developed and NRP extended beyond the sole treatment of HT. However, there are some concerns. For NRP programs to be implemented widely in primary care, a good system with good communication is needed. In the UK, a similar program was introduced in 2003^4^; however, there were concerns about the risk to patient safety, legal considerations, lack of competency, and lack of definition of the role of the healthcare professional in patient care^6^. Physicians may concern about the erosion of their traditional roles and professional hierarchies. There is also a need for the continuing education of nurses and pharmacists in the program^4^. Importantly, for NRP clinics to function, limited prescription rights might need to be granted to trained professionals in Hong Kong. As consultation times in NRP clinics may be longer, their cost-effectiveness should be assessed. What’s more, despite NRPs helping better manage physician workload, a regular review of stable chronic disease is important because polypharmacy has become increasingly common^25^.

A worrying trend that we noticed was that, although our sample comprised only well-controlled HT patients, a significant proportion of both groups still made >5 additional visits to GOPCs, ≥10 additional visits to private clinics, and around 15% were admitted to AEDs or hospitalized in the last 12 months. It is possible that these patients had unmet needs.

Similar to the global trend, China is facing an increasing healthcare demand of patients with multiple chronic conditions. Such significant demand created a huge burden for physicians in outpatient clinics and caused a restricted consultation^26,27^. As a compensation, patient education and physician-patient decision-making are not optimal. Health-care reform is essential, especially for cities on the east seacoast where they are not only facing an aging population but also the burden caused by urbanization. Successful examples from other countries shown that nurse-led RPs will somewhat reduce the physicians’ burden in chronic disease management in outpatient clinics. But compared with the countries where nurses take a more active role, such as UK, US, Australia and New Zealand, the nurses in Asia have limited function in HT management, e.g., they have no prescription right^28,29^. Furthermore, Chinese patients prefer to ask physicians for advice when making decisions rather than nurses due to some traditional cognition^17,30^. But our study has shown that, at least for hypertensive patients, nurse-led RP was well accepted in an outpatient clinic. Whether such new arrangement of HT management will truly lead to more available time for physicians to handle more complicated cases is still questionable. For instance, in a head count system, physicians have no incentive to spend more time with more complex patients than patients with stable chronic HT. Furthermore, how implementable is NRP in the current health system concerning extension nurses’ function in HT management (right of drug prescription)? Implementation research on these questions is essential.

This study had several limitations. First, the trial included only one center and one registered nurse. The efficacy of interventions, as delivered under tightly-controlled protocols in academic research settings, might not translate to effectiveness in regular medical practice settings^31^. However, we believe this study is the first step in further research that explores if NRP can be widely implemented in China. Second, the trial duration was too short to determine clinical outcomes such as mortality or incidence of cardiovascular outcomes. Instead, surrogate outcomes such as patient satisfaction and blood pressure were used. Future study can investigate the long-term effect of the nurse-led clinic to improve medication adherence and decrease all-cause mortality, as suggested by a recent systematic review^32^.

In conclusion, nurse-led repeated prescription was acceptable, safe, and non-inferior to doctors’ consultation for blood pressure control in patients with controlled hypertension. A future multi-center randomized controlled study is needed to generalize this finding.

## Methods

### Study design

We conducted an one-year, prospective, non-inferiority, randomized two-arm intervention study. Patient outcomes were compared between the intervention (NRP) and control (usual care [UC]) care groups using an investigator-blinded randomized design. Patients in both groups went to a routine consultation at their physician at baseline (0 months) and at the end of the study (12 months). A trained research nurse saw patients in the NRP group at 4 and 8 months, while a physician saw patients in the UC group. Both groups were encouraged to book episodic appointments for acute illnesses

The trial was registered at Center for Clinical Research and Biostatistics (CCRB) Clinical Trials Registry, CUHK on 31 March 2014 with the unique trial number ChiCTR-TRC-14004444.

The Joint Chinese University of Hong Kong – New Territories East Cluster Clinical Research Ethics Committee approved this study (CRE2011-2.489). The present study adheres to the Consolidated Standards of Reporting Trials (CONSORT) 2010 guidelines and the operational principles of the Declaration of Helsinki. The completed CONSORT checklist is provided in supplemental contents. Informed consent was signed by all the participants and their privacy information was protected.

### Participants

Assuming a 20% drop-out rate, we planned to recruit 207 participants per arm to detect a between-group non-inferiority margin of 6.7 mmHg with a one-sided 1.25% alpha-level after Bonferroni correction.

We recruited individuals from a government-funded GOPC in Shatin, a large suburb of Hong Kong, from March 28, 2014, to January 30, 2015, through referrals from 15 primary care physicians. Shatin has 630,273 residents whose sociodemographic characteristics (e.g., median age; proportion of individuals with Chinese ethnicity, post-secondary education, and working people) resembles that of Hong Kong^33^. We included individuals who were >18 years of age, were diagnosed with HT, had no changes in their drug prescriptions in the previous 12 months, had a clinic-measured SBP <140 mmHg and DBP <90 mmHg at recruitment, had no history of cardiovascular diseases or diabetes mellitus (DM), and had no HT-related complications as assessed by annual blood and urine checks. Those who were unable to give consent, were concurrently a subject in another clinical trial, planned to be pregnant within the coming year or were pregnant at recruitment, or had a known history of renal impairment or cardiovascular disease was excluded.

### Randomization and masking

Participants were randomly assigned to the intervention or control groups 1:1, using computer-generated random numbers by a statistician who was not part of the research team to ensure allocation concealment. The study design did not allow for blinding of participants from treatment. Clinic assistants were masked to the participants’ allocations. The allocation was unknown to participants until their first appointment.

### Nurse-led repeat prescriptions (intervention group)

A registered nurse was appointed and trained by the investigators. Like in UC, the nurse offered consultations to all NRP participants. To ensure consistency of the NRP sessions, drug compliance and BP were checked at every visit. The pre-developed HT management protocol was designed to be comparable to usual physician practice: if the BP was normal, an RP that was pre-signed by the case physician was issued. If the SBP was >140 mmHg and/or the DBP >90 mmHg, BP was rechecked after at least 10 minutes of complete rest. If the repeat BP was between 140/90 mmHg and 160/95 mmHg, medication was prescribed, but a shorter follow-up visit (in 1 instead of 4 months) was arranged; if the patient had an abnormal BP on the subsequent visit, the nurse consulted the physician. If the repeat BP was a SBP >160 mmHg or DBP >95 mmHg, the nurse consulted the physician within the same day. The nurse was allowed to consult the attending physician regarding complications, side effects, and concerns regarding drug treatment.

### Usual care (control group)

As usual care in Hong Kong, HT patients’ have their BP being measured by a clinic healthcare assistant in the same manner as the NRP group, followed by a physician consultation and drug prescription.

### Outcomes

#### Primary outcomes

SBP and DBP measured at 12 months after randomization.

#### Secondary outcomes

The original version of the Patient Enablement Instrument (PEI) was developed in United Kingdom to assess the enablement of patients after a consultation with their primary care clinician^34^. It is intended to be a measure of the effectiveness of primary care consultations and it has been a widely accepted tool validated in several studies^35–38^. PEI consists of 6 questions (able to cope with life, able to understand your illness, able to cope with your illness, able to keep yourself healthy, confident about your health and able to help yourself) about a patient’s enablement over the preceding 12-month period, with a total score of 0 to 12 and higher scores indicating greater enablement. Each question has four response options: much better/better (Questions 1–4) or much more/more (Questions 5–6) or same or less or not applicable. The Chinese PEI has been validated in primary care in Hong Kong^39^.

Information on the participants’ health service utilization in the past 12 months was retrieved from the CMS. CMS record frequency of consultations at the GOPC and specialist out-patient clinics (SOPC), number of admissions to the accident and emergency departments (AED), and number of hospital admissions. A questionnaire assessed Self-reported visits to private clinics at the 12-month visit.

To assess changes in prescriptions, prescriptions for five anti-hypertensive medications were recorded at baseline and the end of follow-up: Angiotensin-converting enzyme inhibitors or Angiotensin II receptor blockers (lisinopril, Losartan); Beta-blockers (Atenolol, metoprolol); Calcium channel blockers (amlodipine, Nifedipine); Drugs containing thiazide diuretics (Indapamide, Hydrochlorothiazide, Moduretic); and ‘other’ prescriptions (e.g., alpha-blockers or central acting agents such as methyldopa and Prazosin). A dummy variable was created to indicate changes in prescriptions (1 = change of prescription, 0 = same prescription).

Medication compliance was assessed with the 8-item medication adherence scale (MMAS-8), which has been validated in outpatient settings among Chinese patients with HT with excellent reliability (Cronbach’s alpha coefficient 0.83)^40–42^. MMAS-8 scores range from 0 to 8, with higher scores signifying better drug compliance^43^.

### Semi-structured qualitative interviews

To better understand the participants’ experiences with NRP and to explain our quantitative data, semi-structured interviews were conducted with a few subjects in the NRP group at their last clinic consultation. Based on previous literature and the aim of this analysis, an interview guide was developed (eTable 1). Interviews were audio-recorded, transcribed, and coded thematically. Interviews were conducted until the saturation of data, which was defined by no new code or theme in the last batch of transcripts. Two researchers independently cross-checked the coding to ensure reliability. An interview guide was developed by the research team after preliminary analysis of quantitative data and piloted in four participants with HT before use in study participants. The themes used in the interviews included general knowledge on HT, perceptions on NRP, experience with the nurse consultation, and overall evaluation of the NRP program.

### Statistical analysis

Baseline characteristics (age, sex, marital status, educational background, medication use, and history of chronic conditions) were compared using two-sample t-tests for continuous variables and the Chi-square test for categorical variables. The effects of NRP on the primary outcome were examined using ANCOVA with BP as the baseline and treatment group as the covariate, following the intention-to-treat (ITT) principle. Sub-group analysis will be conducted if any characteristic at baseline is unbalanced between two groups. From retrospective primary care clinical data, the estimated SBP and DBP averages in patients with HT were 131.4 mmHg and 74.0 mmHg, respectively. Based on the results from a meta-analysis on BP lowering for prevention of cardiovascular events^44^, and the principle of having half of the mean effect size of previous trials or meta-analysis^45^, we used 6.57 mmHg and 3.70 mmHg as the non-inferiority margins for SBP and DPB, respectively. We accepted the non-inferiority of NRP to UC (in a 0.0125-level test after Bonferroni correction for multiple testing) if the lower bound of the two-sided 95% confidence interval (CI) (equivalent to the upper bound of the one-sided 97.5% CI) was within the non-inferiority margin. We performed equivalence tests for secondary outcomes. We conducted both, modified ITT (mITT) and per-protocol (PP) analyses. PP analysis provides some protection for theoretical increases in the risk of type I error (erroneously concluding non-inferiority)^46^. Our mITT data comprised all patients according to and included in the random allocation of complete data. We defined the PP population as participants who met the mITT definition but never received the intervention treatments.

## Electronic supplementary material

Supplementary Information
