# Supplementary material for: Nurse-led hypertension management was well accepted and non-inferior to physician consultation in a Chinese population: a randomized controlled trial
Source: Sci Rep. 2018 Jul 9;8:10302. doi: 10.1038/s41598-018-28721-2 (PMC6037742; doi:10.1038/s41598-018-28721-2)
Supplement: Supplementary file 1 — Supplementary Information [file 41598_2018_28721_MOESM1_ESM.pdf]

**SUPPLEMENTARY INFORMATION**

# Nurse-led hypertension management was well accepted and non-inferior to physician consultation in a Chinese population: a randomized controlled trial

Benjamin Hon Kei Yip[a,^], Eric Kam Pui Lee[a,^], Regina Wing Shan Sit[a], Carmen Wong[a], Xue Li[a], Eliza Lai Yi Wong[a], Martin Chi sang Wong[a], Roger Yat Nork Chung[a], Vincent Chi ho Chung[a], Kenny Kung[a], Samuel Yeung shan Wong[a,*]

[a]JC School of Public Health and Primary Care, The Chinese University of Hong Kong, Hong Kong SAR, China

^Equal contributors

**Supplemental Digital Content 1**

Theme 1

**(i) Perceived control of HT**

Some participants were happy to be referred to nurse clinic because they perceived that it meant that their HT control was good so that doctors' consultations were not necessary.

*Participants 16: "The first feeling…. So my disease is getting better? So that I do not need to see a doctor"*

*Participants 13: "They said my BP is now stable so that I can see a nurse"*

**Participant 6: "I was happier... I felt my condition (HT) was quite good.**

**(ii) Competency of nurses in HT management**

Participants agreed to follow up in nurse refill clinic because they were referred by their case doctor.

*Participants 5: "If he/she (nurse) allowed to follow up my HT, I am confident that he/she must be capable"*

*Participant 7: "my doctor said I can… it may save my time to see a nurse instead of seeing a doctor"*

(iii) **Additional support from nurses**

Patients felt the care provided by doctors or nurses were similar. The main role of doctors in their HT management was drug management, which was then carried out by the nurse.

*Participant 10: "They (doctors and nurses) gave me good advice, reminding me to take my drugs and monitor my BP."*

*Interviewer: "So they are identical?"*

*Participant 10: "yes"*

*Interviewer: "But will you choose to see doctors more than nurses?"*

*Participant 13: "not really. They are similar."*

**They perceived the main role of doctors were to prescribe HT medications only.**

*Participants 5: "The doctor did not communicate much with me… just prescribe the medications…"*

Theme 2

**(i) Better communication with nurse**

Participants felt they could ask more questions and receive more advice when being followed up by the nurse.

*Participant 5: "I would rather pay more to see a nurse. She was more polite than doctor. I* **could ask more. She said exercise was good for me and I should to avoid salty food. If I can choose, I will** *choose the nurse so that I can ask more questions that concern me."*

*Participant 15: "The nurse asked more details (about HT) than doctors, possibly* **she had more time** *and doctors were busier."*

*Participant 16: "The nurse talked more with me, tried to understanding my life. Doctors concentrated* **more on my illness. They arranged the next follow up, blood tests and such. I felt closer (with the nurse), w***ho talked more and understood more."*

**(ii) Participants felt more relaxed when seeing a nurse**

Participants felt they were more relaxed during nurse consultations.

*Participant 16: "seeing a nurse will make you feel more relaxed… as if my disease is not so serious"*

*Participant 11: "Nursing consultation was more like chatting, which made me more relaxed. I got familiar with the nurse and she concerned about me. We chatted and we talked about my family."*

**Participant 15: "I am always nervous when I go to clinics. Seeing a nurse make me more relaxed."**

**(iii) Better time management**

Participants found that the waiting time before consultations was shorter for nurse refill clinic.

*Participant 16: "Saved time. On average 45 minutes (each visit)"*

They also found that the consultation time with nurses was longer. Short consultation time in doctors' sessions hindered communication.

*Participant 15: "I have more time with the nurse than when I was with my doctors. Doctors need to* **take care of too many patients. Time was so limited that they cannot answer many of your questions.** *A few doctors refused to answer. I dare not ask questions when I see a doctor"*

Some participants hoped that nursing refill clinic will save the doctors' time, which can be useful for patients in need

*Participant 15: "It may ease doctors' work pressure. I think it is ok"*

*Participant 16: "If I can follow up here, it means that my disease is stable. I think it can save time so that other patients in need can see a doctor."*

Theme 3:

*Participant 10: "I want to join the nursing clinic. Better till the end of my life"*

*Participant 11: "If sudden problems arise, the nurse should ask the doctor what to do. If annual complications screening is negative, it is good to see a nurse."*

*Participant 16: "It is ok…* **may be seeing the doctor once then seeing the nurse twice (for follow up). I will** *be more relaxed if there is communication between the nurse and doctor"*

Participants were willing to pay the same (participant 5, 10, 11, 15), or higher price (6, 7,13) for nursing clinic as doctors' consultation

*Participant 7: "HK$50 (standard price for a doctor consultation was HK$45). It is ok to be a bit more expensive than doctor's consultation. I can learn more about HT in the nursing clinic*

Most participants were willing to find alternate medical help if other health problems arise because drugs cannot be prescribed by nurses in Hong Kong.

*Participant 5: "It's ok. We cannot force the nurse to prescribe other drugs. When needed, I will see a private doctor."*

**Supplemental Digital Content 2 - Table**

**eTable 1** Interview guide

| Theme | Semi-structured interview questions |
|---|---|
| General HT information | How were you diagnosed with HT? |
| | How did you self-manage your HT? |
| Recruitment to Nursing clinic | What were your thoughts or feeling when you were randomized to see a nurse instead of doctor? |
| | What is the difference in role between a doctor or a nurse? |
| | What do you expect from the nurse? |
| Experience with the nurse | How did you benefit from the nursing clinic, if any? |
| | What were the disadvantages in seeing a nurse, if any? |
| | Did you have any complaint about the program? |
| | What would you do if you suffered from concurrent medical problem but the nurse could only prescribe you with anti-HT drugs? |
| Overall evaluation of the program | Do you want to continue seeing the nurse if you are given the chance? |
| | What is the amount of money you are willing to pay for the nursing clinic? (Standard doctor consultation fee was 45$/consultation) |
| | Do you think the program is feasible in HK primary care? |

**eTable 2** Retrieved themes from the quantitative analysis

| | | |
|---|---|---|
| *Theme 1: Reasons for high acceptance of NRP* | **(i) Perceived control of HT**<br>Some participants were happy to be referred to nurse clinic because they perceived that it meant that their HT control was good so that doctors' consultations were not necessary. | *Participants 16: "The first feeling…. So my disease is getting better? So that I do not need to see a doctor"* |
| | | *Participants 13: "They said my BP is now stable so that I can see a nurse"* |
| | | *Participant 6: "I was happier... I felt my condition (HT) was quite good.* |
| | **(ii) Competency of nurses in HT management**<br>Participants agreed to follow up in nurse refill clinic because they were referred by their case doctor. | *Participants 5: "If he/she (nurse) allowed to follow up my HT, I am confident that he/she must be capable"* |
| | | *Participant 7: "my doctor said I can… it may save my time to see a nurse instead of seeing a doctor"* |
| | **i) Additional support from nurses**<br>Patients felt the care provided by doctors or nurses were similar. The main role of doctors in their HT management was drug management, which was then carried out by the nurse | *Participant 10: "They (doctors and nurses) gave me good advice, reminding me to take my drugs and monitor my BP."*<br>*Interviewer: "So they are identical?"*<br>*Participant 10: "yes"* |
| | | *Interviewer: "But will you choose to see doctors more than nurses?"*<br>*Participant 13: "not really. They are similar."* |
| | | *They perceived the main role of doctors were to prescribe HT medications only.*<br>*Participants 5: "The doctor did not communicate much with me… just prescribe the medications…"* |
| *Theme 2: Interaction with the nurse* | **(i) Better communication with nurse**<br>Participants felt they could ask more questions and receive more advice when being followed up by the nurse. | *Participant 5: "I would rather pay more to see a nurse. She was more polite than doctor. I could ask more. She said exercise was good for me and I should to avoid salty food. If I can choose, I will choose the nurse so that I can ask more questions that concern me."* |
| | | *Participant 15: "The nurse asked more details (about HT) than doctors, possibly she had more time and doctors were busier."* |
| | | *Participant 16: "The nurse talked more with me, tried to understanding my life. Doctors concentrated more on my illness. They arranged the next follow up, blood tests and such. I felt closer (with the nurse), who talked more and understood more."* |
| | **(ii) Participants felt more relaxed when seeing a nurse**<br>Participants felt they were more relaxed during nurse consultations | *Participant 16: "seeing a nurse will make you feel more relaxed… as if my disease is not so serious"* |
| | | *Participant 11: "Nursing consultation was more like chatting, which made me more relaxed. I got familiar with the nurse and she concerned about me. We chatted and* |

| | | | |
|---|---|---|---|
| | | | *we talked about my family."* |
| | | | *Participant 15: "I am always nervous when I go to clinics. Seeing a nurse make me more relaxed."* |
| | **(iii)** | **Better time management**<br>Participants found that the waiting time before consultations was shorter for nurse refill clinic. | *Participant 16: "Saved time. On average 45 minutes (each visit)"* |
| | | | They also found that the consultation time with nurses was longer. Short consultation time in doctors' sessions hindered communication.<br><br>*Participant 15: "I have more time with the nurse than when I was with my doctors. Doctors need to take care of too many patients. Time was so limited that they cannot answer many of your questions. A few doctors refused to answer. I dare not ask questions when I see a doctor"* |
| | | | Some participants hoped that nursing refill clinic will save the doctors' time, which can be useful for patients in need<br><br>*Participant 15: "It may ease doctors' work pressure. I think it is ok"*<br><br>*Participant 16: "If I can follow up here, it means that my disease is stable. I think it can save time so that other patients in need can see a doctor."* |
| *Theme 3: Value of the NRP clinic* | **(i)** | **Patients wished to continue follow up in the NRP** | *Participant 10: "I want to join the nursing clinic. Better till the end of my life"* |
| | | | *Participant 11: "If sudden problems arise, the nurse should ask the doctor what to do. If annual complications screening is negative, it is good to see a nurse."* |
| | | | *Participant 16: "It is ok… may be seeing the doctor once then seeing the nurse twice (for follow up). I will be more relaxed if there is communication between the nurse and doctor"* |
| | **(ii)** | **Participants were willing to pay the same (participant 5, 10, 11, 15), or higher price (6, 7,13) for nursing clinic as doctors' consultation** | *Participant 7: "HK$50 (standard price for a doctor consultation was HK$45). It is ok to be a bit more expensive than doctor's consultation. I can learn more about HT in the nursing clinic* |
| | **(iii)** | **Most participants were willing to find alternate medical help if other health problems arise because drugs cannot be prescribed by nurses in Hong Kong**. | *Participant 5: "It's ok. We cannot force the nurse to prescribe other drugs. When needed, I will see a private doctor."* |

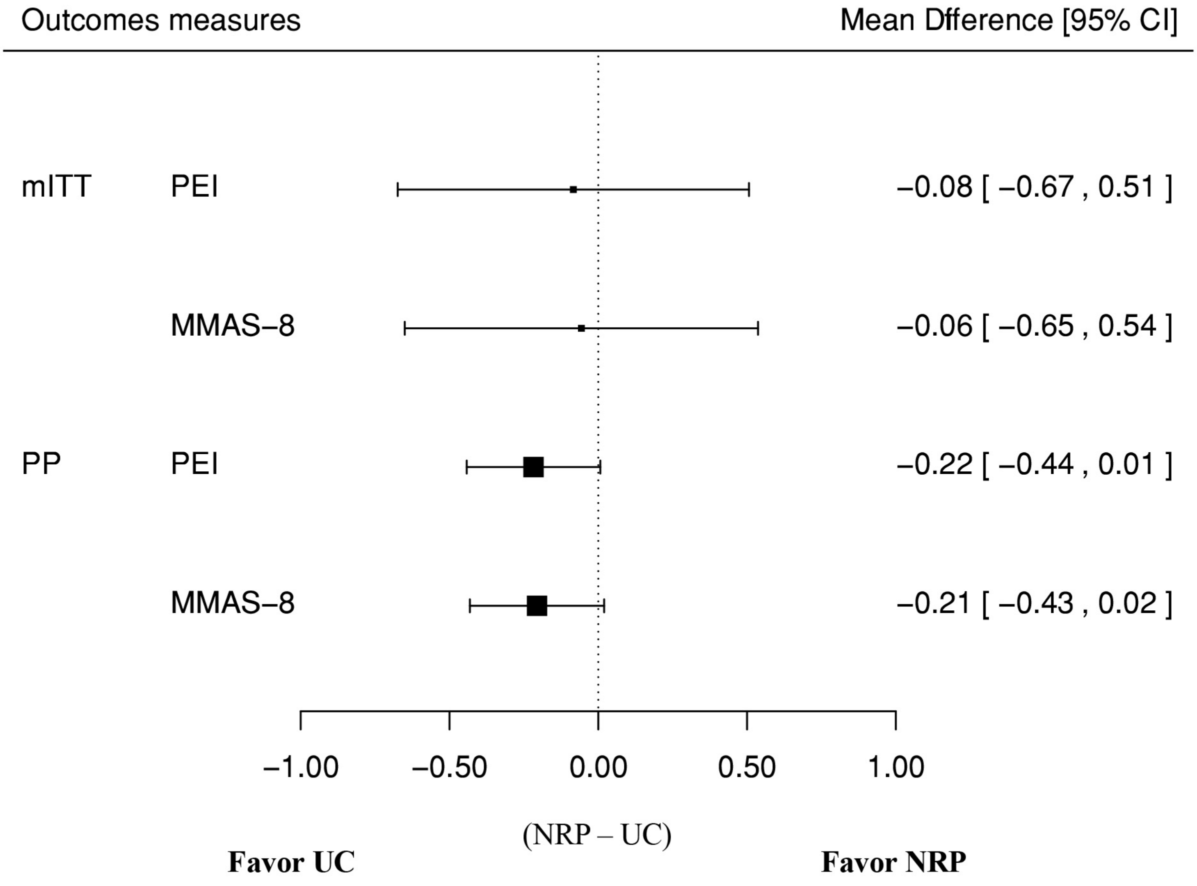


**eFigure 1** Estimated mean difference in Patient Enablement Index (PEI) and medical compliance (MMAS-8) between intervention group (nurse led repeated prescription, NRP) and control group (usual care, UC), by modified Intention-to-treat (mITT) and per-protocol (PP) analysis.
